# Gender Perspective on Older People's Exercise Preferences and Motivators in the Context of Falls Prevention: A Qualitative Study

**DOI:** 10.1155/2018/6865156

**Published:** 2018-07-18

**Authors:** Marlene Sandlund, Petra Pohl, Christina Ahlgren, Dawn A. Skelton, Anita Melander-Wikman, Birgitta Bergvall-Kåreborn, Lillemor Lundin-Olsson

**Affiliations:** ^1^Department of Community Medicine and Rehabilitation, Physiotherapy, Umeå University, Umeå, Sweden; ^2^School of Health and Life Sciences, Institute of Applied Health Research, Glasgow Caledonian University, UK; ^3^Department of Health Sciences, Health and Rehabilitation, Luleå University of Technology, Luleå, Sweden; ^4^Department of Computer Science, Electrical and Space Engineering, Luleå University of Technology, Luleå, Sweden

## Abstract

**Background:**

Several factors have previously been identified to positively influence the uptake and adherence for fall prevention exercise programmes. There is, however, a lack of studies investigating if men and women differ in their views and preferences for fall prevention exercises.

**Aim:**

To explore exercise preferences and motivators of older community-dwelling women and men in the context of falls prevention from a gender perspective.

**Methods:**

Workshops including multistage focus group discussions were conducted with 18 older community-dwelling people with and without history of falls. Participants were purposively selected and divided into two groups. Each group met on six occasions over a period of five months. Participatory and Appreciative Action and Reflection methodology was used to guide the discussions. A qualitative content analysis approach was used in the analysis.

**Results:**

Older participants had many diverse preferences and confirmed that individually tailored exercise, in terms of mode, intensity, challenge, and social context, is important. Moreover, important factors for exercise adherence and maintenance included the experience of individual confirmation; different spirit lifters to increase enjoyment; and personal tricks to maintain exercise routines. The individual differences within genders were more diverse than the differences between women and men.

**Conclusion:**

Exercise interventions to prevent falls should be individually tailored, based on the specific needs and preferences of the older participant, and do not appear to require gender specific approaches. To increase adherence, intrinsic motivation for exercise may be encouraged by competence enhancing confirmations, energizing spirit lifters, and practical tips for exercise maintenance. The study provides an awareness about women's and men's preferences for fall prevention exercises, and this information could be used as guidance in designing inclusive exercise interventions.

## 1. Introduction

Globally, accidental falls are a major public health problem and the second leading cause of unintentional injury deaths worldwide. Each year 37.3 million falls require medical attention, and adults older than 65 years of age suffer the greatest number of fatal falls [1]. At least one-third of community-dwelling people aged 65 years and above fall every year, and the incidence increases with advancing age and fragility level [2]. In Sweden, falls are the most common cause of injury in old age that leads to deaths, hospitalizations, and visits to emergency services. In the age group over 80 years, nine out of ten injuries are caused by a fall [3]. As the proportion of older people is rising globally, the individual and societal costs associated with falls will increase. The prevention of falls is therefore an urgent public health challenge.

Intervention programmes that focus on balance combined with muscle strength in the lower limbs are effective in addressing risk and rate of falling [4, 5]. Despite this, many older people are reluctant to adopt fall prevention exercise due to factors such as underestimation of falls risk, poor self-efficacy, fear of falling, or stigma associated with exercise for older people [6]. According to a systematic review, only 64% accepted the invitation to join the exercise programme and 19% then dropped out when they learned what the intervention entailed [7]. Moreover, around 77% of the participants in fall prevention exercise studies are women [8]. Instead of participation in exercise, many older people choose individual coping strategies, tailored to their particular circumstances, to avoid future falls, or simply discontinue activities that would involve a risk of falling [9]. Poor adherence is associated with low education (< high school), living in a disadvantaged neighbourhood, being obese, having fair/poor self-rated health and having problems with walking or using a walking aid, and a history of falls [10, 11].

Several factors have been identified that positively influence the uptake and adherence for fall prevention exercise programmes, including facilitators such as support from professionals or family, social interaction, perceived benefits, a supportive exercise context, feelings of commitment, and having fun. However, there is still a lack of studies investigating preferences for specific programme characteristics that may attract older people by making exercise interesting, challenging, and enjoyable [12]. Additionally, in previous studies investigating preferences, more than 75% of the participants have been women and very few studies have investigated if men and women differ in their views and preferences for fall prevention exercises [12]. Unconscious preunderstandings and expectations of how older men and women would prefer to exercise may lead to unnecessary gender bias [13]. In order to identify factors that attract both older women and men to fall prevention exercise programmes, as well as increasing the awareness and knowledge among professionals who deliver these exercise programmes, there is a need to identify if older women and men differ in their views and preferences about exercising. The aim of this study was, therefore, to explore exercise preferences and motivators of older community-dwelling women and men in the context of falls prevention, from a gender perspective.

## 2. Methods

This study is based on workshops including multistage focus group discussions [14] held with older people as part of a larger participatory action research project, aiming to develop an evidence-based, cocreated, fall prevention digital exercise programme [15]. The checklist from the 32-item COREQ (consolidated criteria for reporting qualitative research) [16] was used when designing and reporting the study. The study was approved by the Regional Ethical Review Board (Dnr. 2012-170-31M).

### 2.1. Participants and Recruitment

Participants were recruited from seven senior citizen organisations. Thirty-eight older adults volunteered to be contacted by telephone and interviewed to inform a purposive sample consisting of community-dwelling women and men of at least 70 years of age. Sampling aimed to achieve a heterogeneous group with respect to exercise habits, living conditions, and experiences of falls, with 30% having a fall in the previous 12 months (reflecting population epidemiology). Eighteen participants (10 women and 8 men) were finally included in the study; one woman chose to end her participation after four meetings. The mean age of the participants was 74.6 ± 3.5 years. The majority of the participants were former white-collar workers and, in general, have self-reported being moderately physically active. Two couples were deliberately involved in the project. Four participants lived alone and 14 lived with partners (Table 1).

The participating researchers formed a cross-disciplinary group from the disciplines physiotherapy, informatics, exercise physiology, and computing sciences. The group included experts in e-health, falls prevention, and gender research. All researchers were women. None of the researchers had any preexisting relationship with the participants and they all introduced themselves and their role in the project at the beginning of the process.

Prior to the first meeting, the participants were divided, by the researchers, into two separate groups with similar disposition regarding age, background, and gender. One of the married couples was placed in each group. Before the first focus group discussion, the participants signed a written informed consent.

### 2.2. Data Collection

Data was collected through six workshops including multistage focus group discussions [14] with each group. Each workshop was held at a community centre and lasted for 2.5 hours, including a short refreshment break. All focus group discussions were conducted in a positive atmosphere inspired by Participatory and Appreciative Action and Reflection (PAAR) methodology [17]. The PAAR methodology is a form of Appreciative Inquiry, meaning that it focuses on positive experiences rather than problems. According to this methodology the focus group discussions aimed to understand the root causes of the older participant's positive experiences of and preferences for exercises. The focus was on accomplishments, strengths, and understanding the causes of success, so that these causes could be better understood and augmented. In this way the discussions were not preoccupied by problem solving or by changing negative behavior. This appreciative form of inquiry aims to create an open, positive, and encouraging climate during the discussions.

In order to stimulate the discussions within the group, a range of facilitating techniques and methods were used. The participants were asked, for example, in between the workshops to document their everyday life in different ways in order to stimulate and trigger thoughts about what inspired them to exercise and to be active. One method used for this was Photovoice, a process by which people can identify, represent, and enhance their thoughts through photographs [18]. Another example was to bring pictures from newspapers, reflecting their attitudes towards exercising and moving their body. During other sessions practical activities (e.g., strength and balance exercises) were conducted and the participants were asked to actively reflect over the exercises and to describe their views and feelings. Sometimes more detailed questions were posed and visualisations of different views were placed around the room. The participants were asked to move to the place that represented their view and were encouraged to explain and discuss their choices. Another facilitating technique used was short descriptions of five fictional characters (personas) representing different motivational profiles [19]. These personas were constructed based on data from the first sessions and the participants rated how well the descriptions defined themselves and the way they preferred to do exercises. The participants were later asked to create their own persona description, by combining sentences from the predefined examples. With the purpose of clarifying and separating differences in the participants reasoning, smaller gender divided discussion groups were organized during the fourth and fifth workshop. The main activities of each workshops have been presented elsewhere [15, 20].

Between five and six of the researchers attended each workshop, except workshop three when only two researchers participated (PP and LLO). Two of the authors (BBK and AMW) had extensive previous experience from participatory action research and they were primarily responsible for facilitating the discussions, assisted by the other authors present. After each session the researchers discussed and reflected on the results and the process. Based on these reflections the purpose of the next meeting was planned. In the beginning of each new meeting the researchers summarized and presented result from the last workshop to get feedback, and then the aim of the current meeting was outlined.

### 2.3. Analysis

All focus group discussions were digitally recorded and transcribed verbatim. As recommended when trying to identify similarities and differences in material, qualitative content analysis was used to analyse data and explore women's and men's reasoning about their experiences and preferences in relation to exercises [21]. An inductive content analysis approach was used since very few previous studies have investigated if men and women differ in their views and preferences for fall prevention exercises. Thus the categories were derived from the interviews and not on the basis of previous knowledge or theories [22]. Two of the authors (PP and MS) read the transcripts independently to get an overall understanding of the participants exercise preferences. The text was then transferred to the data program Open Code 4.01 [23], where meaning units related to the aim were identified and labelled with codes. The codes were organized into preliminary subcategories and categories by two of the authors (PP and MS). The categories were continuously negotiated and the texts reread within the author group until consensus was reached to ensure trustworthiness. The categories and subcategories were in addition presented and discussed at two seminars for PhD students and senior researchers at the University. In the end of this process the results were summarized and presented to the participants. Preliminary results were included in the thesis of one of the authors (PP) [20].

## 3. Results

**Figure 1:**
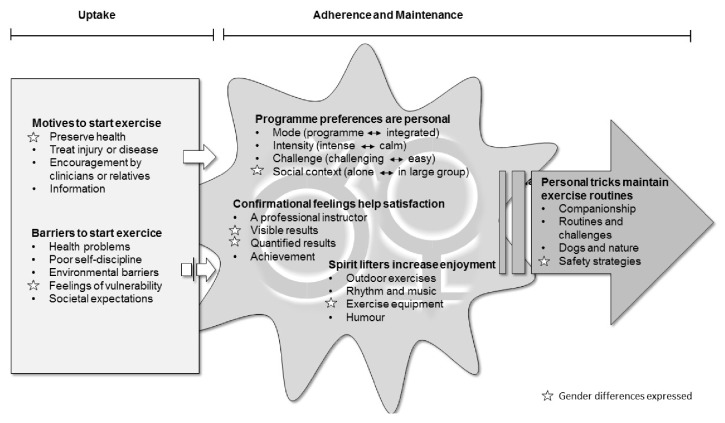
Overview of the categories related to uptake, adherence, and maintenance.

The participants gave many examples of physical activities they liked to perform, such as walking, cycling, working outdoors, gymnastics, water aerobics, skiing, dancing, Nordic walking, table tennis, and Qigong. They expressed many motives, as well as barriers, for starting to do exercise in the context of falls prevention. Based on their experiences, they voiced many opinions regarding how different exercise properties influence and enhance motivation and they gave examples of “tricks” to facilitate maintenance. In the majority of these opinions no clear gender separation pattern was seen. The individual differences within genders were more diverse than the differences between women and men. The results are presented in six categories with subcategories. Two categories were related to* uptake *and four categories concerned* adherence and maintenance*. An overview of the categories and subcategories is illustrated in Figure 1. Quotations within the text are annotated with participant number and the sex of the participant (W or M).

### 3.1. Uptake

Several factors important for uptake were mentioned in the focus group discussions. Some of them were predominantly expressed as* motivators* and others as* barriers* to starting exercise.

#### 3.1.1. Motivators and Barriers to Starting Exercise

The motives for starting to exercise, discussed within the groups, were preserving health; treating injury or disease; encouragement by clinicians or relatives; and information. Common barriers were health problems; poor self-discipline; environmental barriers; feelings of vulnerability; and societal expectations.

Keeping healthy was a major motivator for both men and women. However, there were differences in how men and women expressed their reasons for this. Women talked about preserving health as a necessity in order to manage their responsibilities in their everyday life “*I just have to keep on going myself, so It's just that I need to exercise for my survival*” (W2), whereas men talked more in terms of keeping fit* “You notice that you have to do certain things to… keep fit” *(M1). An injury or disease that could improve by continuous exercise (such as being diagnosed with osteoporosis, sustaining fractures, having a rupture of the Achilles tendon, or suffering from heart disease) was mentioned by both sexes as a motivator for starting to exercise. On the other hand, commonly occurring health problems, such as arthritis and pain, were also major barriers for exercising and sometimes the participants needed to adapt their activities to their health situation.

Professional advice from a doctor or other health personnel was seen as a driving force for starting to do exercises. For some this was also linked to expectations of what it means to be a good patient, as expressed by one of the men in the study “*When you know that you have done what you promised to do, you feel satisfaction in not misbehaving*” (M5). Relatives' influence, for example, persuasion of a spouse or a wish to do certain activities with grandchildren, was also a motivator for being physically active in the context of falls prevention that was frequently mentioned by both women and men. While information in the media inspired some of the participants to exercise:* “I have started to do an exercise programme that works on strength. I saw it on a TV-show”* (W9), other participants said that such information only stayed in their memory for a short time period and seldom resulted in long-term changes.

A common barrier, articulated by both women and men, was poor self-discipline, even though they knew they felt better once they got started* “You have to overcome certain thresholds in order to get out and exercise” *(M4). Environmental barriers, such as the weather, icy or snowy roads, were also common and even though many of the participants liked exercising outdoors, they felt they were less active during the winter. Feelings of being vulnerable or fragile were also expressed as barriers to physical activity by both men and women. However, experience of fractures and fear of falling were particularly experienced as a barrier among the women and caused them to be more cautious in their activities.* “Of course if you have suffered fractures a few times you are very scared after that”* (W9). The men did not generally speak about fear or vulnerability, but many of them noticed that they had started to avoid doing heavy tasks, if possible, as this often led to pain or discomfort in the body for days afterwards. Other examples of situations where feelings of vulnerability or embarrassment could pose a barrier for taking part in exercises were when an exercise group was too small or predominantly one gender, such as being the only man in a group* “I thought I would do it… but I, I dared not *(laughs),* I'd be alone. But then a few men showed up and eventually I dared to pull myself down into the water” *(M1).

The participants expressed different views regarding society's perception of older people. Opinions emerged that older people were often illustrated in media as passive individuals, which may affect older people's views of what is expected of them. “*Well, it is not easy to find any pictures in newspapers illustrating active older people doing gymnastics and such. There is almost nothing!*” (W9). In contrast, other participants expressed the view that older people nowadays are seen as active and that they have more opportunities and resources than older people in former generations.

### 3.2. Adherence and Maintenance

The categories summarizing factors of importance for adherence and maintenance that emerged in the analysis of the focus group discussions were as follows:* programme preferences are personal; confirmational feelings help satisfaction; spirit lifters increase enjoyment; *and* personal tricks maintain exercise routines.*

#### 3.2.1. Programme Preferences Are Personal

Above all the older participants expressed that the exercises need to be adapted to the individual in order to stimulate adherence. When it comes to how the older participants preferred to do their training, in terms of* mode*,* intensity*,* challenge*, and* social context*, no general or gender specific patterns were discerned. Instead the discussions pointed to great variation between individuals, and sometimes even within an individual, depending on the situation.

Regarding exercise mode, diverse preferences for exercise to be either conducted as a programme on specific occasions, or integrated in everyday activities were expressed. Those who were more accustomed to exercising seemed to prefer training programmes, while the more inexperienced felt that integrated exercise was preferable.

The preferred degree of intensity also varied between individuals with no gender pattern; some did not like to sweat, while others thought exercising was not worthwhile if they did not feel tired and sweaty afterwards. What was emphasized was the importance of being able to hold an exercise tempo of one's own. Not being able to keep up or being prevented from exerting effort in their own style and tempo could be frustrating. “*But, we *(with husband)* can almost not walk together *(laughs)*… you must have your own pace… if one of you is faster, and one is… well….”* (W3).

Many of the participants took part in group training and did not seem to worry about the level of challenge, e.g., not being able to keep up or perform all the elements correctly. However, they felt that the instructors should emphasize that if someone is not capable of performing a specific exercise, it is acceptable to choose an alternative exercise, as long as one continues to be active. One man even expressed that it may be motivating to see that there are more challenging levels to strive for, even if you currently cannot perform them. “*But I think it may actually be stimulating when you can see that there are different levels of challenges” (M4).*

Some of the participants liked to work out alone while others preferred to exercise together with others. However, such preferences regarding the social context could vary depending on the activity in focus. When exercising in a group setting, a moderate group size composed of peers of the same age was preferred and the social aspect were emphasized as an important motivator.* “I think that the social aspect is very important in group training… because you usually attend at specific sessions and you meet the same people. Then you start to talk to each other and you learn to know each other… that's fun” (W4). *No obvious gender pattern in preferences for the social context when exercising emerged during the focus group discussions. However, in the personas descriptions constructed by the participants to describe their own motivation profile, women were more prone than men to choose sentences representing a social component to describe themselves, such as “*I am social*”, “*I like to exercise together*”, or “*I prefer to work out in a group*”.

#### 3.2.2. Confirmational Feelings Help Satisfaction

Confirmation of the effectiveness and efficiency of an exercise were important motivational drivers for both sexes.* “You want your efforts to pay off”* (W3). Confirmation could be perceived in different ways: from a* professional instructor*; through* visible results*; by* quantified results*, or merely by the feeling of* achievement*.

To have a professional and encouraging instructor was considered very important. The participants appreciated having an expert to trust and ask for advice. Their encouragement and praise could be very stimulating. Participants expressed that they felt more inspired by an instructor of the same age, someone they could identify with, while the sex of the instructor did not matter at all. “*Yeah, but… the closer to one's own age, I think that gives more*” (M3).

Both women and men liked to achieve a secondary goal through their training which meant that they often integrated training with everyday activities to get a visible results, such as working in the garden, cleaning their home, or going out for a walk with a clear purpose in mind. One man expressed that he did not want to* “…fool around doing peculiar exercises”*.* “With my age… I do not want to just stand and… I prefer to pay attention to my everyday life and then try to integrate my exercises”* (M2). However, a pattern emerged in the participants' personas descriptions suggesting gender differences. It was more common for men to choose expressions representing visible and practical secondary goals, such as “*I want to see a concrete result with benefits*”, “*I weave my exercises into everyday activities*”, or “*I like working in the garden or my home*”.

Many of the participants expressed that quantifying the results of their training was motivating. They wanted to keep track of their performance and see objective outcomes. Some of them used instruments for counting steps and calorie expenditure, or tracking walking or cycling routes etc., and this was perceived as stimulating. Men tended to express more fascination with opportunities to quantify their performance, as this conversation between two men illustrates “*… And then I start to walk again and it's activated, it measures my speed, and how far I've walked and how many calories I have been spent” *(M5). “*Interesting!” *(M7 comments).* “Yes, fun!” *(M5). However, there were also women who expressed interest in these kinds of devices. Contrastingly, there were also people who seemed to be totally disinterested in measuring their performance and were perfectly happy just knowing that they accomplished the task. One woman described how she had an exercise bike that could record several things but she was not interested in using these features “*I am not doing anything *(to record)*… I sit and cycle for half an hour, and am fine with that” *(W4).

The participants expressed that an inner feeling of achievement was important for adherence to exercise. They associated the pleasant sense of relaxation in the body afterwards, and the pure satisfaction in managing to work out, with an intrinsic motivation for continuing to exercise.* “Well… when you know you have done something, and you can take a shower and sit down in the armchair with a good conscience ” *(M5) or “*You experience the same thing when doing exercises, -relaxation… when you are active, and feel that you actually manage to be active”* (W5).

#### 3.2.3. Spirit Lifters Increase Enjoyment

The focus group discussions revealed the importance of spirit lifters, in an exercise context, i.e., dimensions that could contribute to enjoyment and motivate older participants to continue to be active. These included* outdoor exercises; rhythm and music; exercise equipment*; and* humor*.

Outdoor activities were frequently highlighted as very motivating. Both women and men liked the idea of exercising outdoors. “*I like to be out there… perhaps because I have been sitting inside all… well quite a few years now… it's great to be able to be outdoors*” (W3). Many of the participants engaged in outdoor activities all year around, and some clearly expressed a dislike for exercises indoors. “*I had a gym card when I was employed… I went a few times but I do not get the point… biking indoors when you can bike outdoors and get fresh air?*” (M5). Many of the examples from the participants' outdoor activities referred to taking a walk, cycling, or cross-country skiing. However, at the beginning of the project strength and balance exercises were still mainly performed indoors and only a few participants could imagine doing these kinds of exercises outdoors. During the course of the study though, some of the participants became inspired by the discussions and started to add balance exercises during their regular walks. These participants could later on give examples of suitable ways to do this, which opened the eyes of the other participants to these possibilities.

Another spirit lifter discussed was the use of rhythm and music, which were perceived as engaging and thought of as providing a pleasant atmosphere.* “Yes, music, I like music! Absolutely! For me music is very important when exercising. I know that everyone does not like it, but I think the music means a lot”* (W6). Although the participants generally liked music during their exercises, they highlighted that the music had to be tailored to the participants' age, and the rhythm had to be in line with the exercises performed. Otherwise, the music became frustrating.* “Well, when I do water aerobics… it is often disco music… very rhythmic and the girls *(instructors)* are too darn good and move in time. But they are not in the water…and when I'm not able to do the movements…I become frustrated”* (M8).

The use of exercise equipment was considered effective and a motivating element in an exercise context. However, women often noted that they did not want to buy equipment to use in their own homes; it was better if they got tips on how to use things already available in a household.* “Not to buy a lot of things… I believe it is better to take what you have at hand” *(W1). Men on the other hand preferred specific equipment* “Yes, but those things can sometimes fit … have a better grip and such… it's fun with the real stuff” *(M1). The participants themselves also commented that the use of equipment might be gender related. Furthermore, the participants explained how the use of exercise equipment could contribute in the creation of a social exercise context. For example, both men and women liked to walk with poles and to meet peers who did the same, as this created a feeling of belonging to a group.* “It's nice when you walk with poles, when you go out for an ordinary walk, and you meet unknown people you just walk on, but with poles, then you say hello, it's like you belong to a group in a sense. I think that feels nice*” (M1).

Humor was emphasized as an important source of inspiration. The participants expressed that it should be joyful to exercise. Humor could involve having an instructor joking and teasing, or just giving yourself a chance to enjoy. An exercise session full of laughter and happiness could create a lingering contentment afterwards*. “Something I think is often overlooked is the importance of humor. I think it's very important that you get to laugh”* (M2).

#### 3.2.4. Personal Tricks Maintain Exercise Routines

Several tricks or strategies were mentioned by participants to maintain their exercise routines and to prevent lapses and relapses. These tricks involved* companionship*;* routines and challenges*;* dogs and nature*; and* safety strategies*.

Friends or spouses could push each other “*When you are alone, then you have to put in an active effort to get going… it will be easier to find arguments against …-it looks like bad weather out there! or whatever*” (M1). Having company was also inspiring, “*So… well, she is the one who is pushing. And I think it is much, much nicer if we're together compared to if I am going out alone*” (M4).

The participants gave examples of having routines, such as specific tracks they walked, which helped them to exercise regularly. To set goals, as well as to start with one that felt achievable, was also proposed as a powerful way to get over the threshold when it felt difficult to start, because, once started, it was often easy to continue. “*It may feel tough in the beginning, but if I think, -Nah, but today I will just take a trip up to the road. Then suddenly, it feels pretty good after all, and I walk on and continue for about a full hour. And that feels great, although there were setbacks when I began*” (W9). A competitive element could also be an incentive for both men and women, but the vast majority created challenges solely for themselves. Other participants emphasized that they did not like competitive elements, “*That you are challenging yourself… I think that is just a pain… Then it becomes a must-do that should be avoided… I have done enough of that in my life”* (M4).

Dogs were mentioned as great helpers to maintenance exercise.* “Yes, we've had a dog, and I miss it immensely, for it was the best exercise help I ever had”* (M7). Several of the participants had a dog and felt this kept them active and provided some company. Some of the participants had even taken it upon themselves to go out with someone else's dog, just to get exercise. “*Therefore, I have an extra dog that I go out with… then I have to go out… and then I feel good”* (W3). Even without a dog the pure experience of nature itself could also be an incentive to go for walks. Feeling the freedom, enjoy listening to the birds, and getting fresh air were described as addictive.

Some participants, mainly women, expressed that a fear of falling could inhibit their activity. But they also gave examples of safety strategies they developed in order to feel more secure and thus gave them the confidence to continue their physical activities. One example discussed was walking with poles, to have support on slippery surfaces. Another example was to bring the mobile phone to be able to call someone if and accident should occur. “*Just that using a mobile phone… I always take it with me when I go out for a walk, even though I will not be long, especially if I you are somewhere where no one knows where you are”* (W6).

## 4. Discussion

These multistage focus group discussions aimed to improve our understanding of the preferences community-dwelling older women and men have regarding exercise in the context of preventing falls. Older participants had many diverse preferences and confirmed that individually tailored exercise programmes, in terms of mode, intensity, challenge, and social context, are important. Moreover, important factors for exercise adherence and maintenance included the experience of individual confirmation; different spirit lifters to increase enjoyment; and personal tricks to prevent lapses and relapses. It is interesting to note that no clear gender separation pattern was seen in the majority of these factors (Figure 1).

It may seem obvious that older people, just like younger people, have very different opinions on how they prefer to perform their exercises. But all too often, older adults seem to be considered as a homogenous group and recommended the same kind of training regardless of needs or preferences. Studies from Sweden have, as an example, shown that walking is by far the most suggested physical activity for older people getting exercise prescriptions in health care settings [24], although it is known that brisk walking will actually increase the fall risk in those with a history of falls [25]. Previous studies have also shown that when the prescribed exercises are not tailored to the individual, and seniors with varying degrees of physical capacity are expected to exercise together, they are frustrated and may drop out [26]. Our results emphasize the importance of taking the experiences and desires of the older individual into account when fall preventive exercises are planned and prescribed.

Previous research indicates that women are more likely to take part in exercise activities to prevent falls [8]; in light of our results that is not due to substantial differences in exercise preferences among women and men. However, a recent literature review suggests that both women and men see women as more receptive to and in more need of fall prevention messages [12]. This may be due to societal norms. Our understanding of gender derives from a social constructionist perspective that gender is created in social relations and dependent on time and context [27]. This means that humans, as social beings, shape themselves in relation to prevailing norms in society for how to be a man or a woman and this is referred to as “doing gender” [28]. According to the norms, men should be independent, self-reliant, strong, tough, and willing to take risks [29]. Physical strength, competition, and risk-taking are all characteristics in “hegemonic masculinity” [30]. These masculinity characteristics often preferred among younger men could have been deemphasized with ageing and a changed life situation among the men in this study. The men were retired, some were widowers, and others were carers for relatives. These circumstances may have contributed to the many similarities in preferences expressed by men and women in this study.

Some differences between men's and women's preferences for exercises were expressed in this study; for example, women talked about preserving health, whereas men wanted to keep fit; men preferred exercise equipment in training; men were more prone to emphasize the importance of quantified results as well as having visible secondary goals; women stressed the social component; and the women, but not men, expressed fear of falling as a barrier for exercise. The gendered patterns revealed in this study are not surprising, since they reflect the social gender norm. Nevertheless, these gender differences may be important for professionals to address when “marketing” exercises to prevent falls, in order to attract both men and women.

An understanding of how to support motivation in a specific situation requires an understanding of how activities and contexts are experienced by the older person. The Self-Determination Theory (SDT) is a social cognition model that addresses the process through which a person acquires motivation for initiating a new health-related behavior and maintains it over time [31]. According to SDT, maintenance of behaviors over time requires that the person internalizes the behavior in order to develop an intrinsic motivation. In this process of internalization the reinforcement of three innate psychological needs: autonomy, competence, and relatedness, is critical [32, 33]. For behaviors to be successfully changed and maintained individuals must come to personally endorse their importance and make an autonomous, intentional, and volitional decision to change. The person must also feel competent and confident enough to change. Finally, relatedness feelings of being included and cared for by others in the relevant context are important [32, 33]. These three innate needs are clearly reflected in the categories summarizing how the focus group participants described factors important for adherence and maintenance. By relating the results of this study to the support of psychological needs, we can gain an increased understanding of how to apply key motivating elements in fall prevention exercises for older people.

### 4.1. Uptake

The motives for starting to do exercises (uptake) expressed by the participants in this study were mainly framed in terms of inherently satisfying goals such as to preserve health and to treat injury or disease, which means the participants valued exercise highly and identified with the behavior. A focus on such intrinsic goals (e.g., being healthy) as opposed to extrinsic goals (e.g., being physically attractive) is believed to increase maintenance over time [32]. Receiving support and receiving adequate information were also cited as important factors for taking up exercise to prevent falls. All these motives are in accordance with previously reported preferences for falls prevention exercises [12] as well as for falls prevention programmes in general [6, 34].

The barriers cited as reasons for not participating in exercises were also consistent with previous studies, e.g., health problems or environmental barriers [12] and poor self-discipline and lack of motivation [26, 35]. However, in our previous review, an anxiety about not being able to keep up with demanding exercises was noticed in many articles [12]. In the present study the participants did not seem to worry about performing correctly at all times, as long as they felt assured by the instructor that doing as good as they could was good enough. On the other hand, feelings of vulnerability, such as fear of falling, or being the only man in a group, were expressed as barriers by the participants. According to the SDT one way to help individuals integrate and internalize a behavior is to support exploration of resistances and barriers and help in the identification of possible solutions [32].

### 4.2. Adherence and Maintenance

Based on the results of this study an individually tailored exercise program seems crucial to stimulate adherence and maintenance, which is in accordance with several previous studies [26, 35–38]. Support for the importance of individualization can also be found in the SDT, which suggests that autonomy and self-determination are supported when opportunities for reflection and choices are allowed. A strong sense of autonomy will, in turn, contribute to internalization of values and skills for change, leading to increased intrinsic motivation and consequently also improved exercise adherence [32, 33].

Those participants who were more accustomed to exercise seemed to prefer training programmes, while the more inexperienced felt that an integrated exercise, into their daily life activities, suited them better. These results seem to conform well with the results of a previous study, who described their moderately active participants as “functional exercisers” who favored activities perceived as purposeful and practical, often unplanned and low in intensity [39]. Such integrated exercises, where balance and strength training are integrated in activities of everyday routines, have been proven effective in protecting older high risk people from falling and in improving and maintaining their functional capacity [40]. It may be a promising alternative to traditional exercise programmes for many older people who are not used to exercising or who do not want to exercise in a group. Earlier studies have suggested that low intensity exercises may facilitate participation [6]. However, the participants in this group expressed preferences for both “calm” and “intense” exercise, again reminding us of the need to consider individual preferences.

The importance of social interaction, and group adherence, is often found to be an important factor to enhance participation in exercise among older people [6, 12]. According to the SDT social interaction will naturally contribute to feelings of relatedness, which is important for intrinsic motivation to develop [33]. Social interaction was emphasized as important by some in this study, in particular by women in their own persona descriptions, while some of the participants preferred to exercise alone. Similar findings have been found in a previous study from the UK, in which more women than men were likely to attend group sessions [41]. In another study comparing exercise habits in older people in Sweden and Ireland, the Swedish participants tended to have a higher regard for solitary exercise in comparison to older persons from Ireland, where physical activity was often seen as a means to socialize [39].

To receive confirmation of being able to exercise and that one's efforts actually give results was expressed as a very important element in an inspiring exercise context. Confirmation could be seen through visible results of practical activities, by assessment and evaluation of performance, or simply by feelings of achievement. Confirmation could also be obtained through feedback from a professional instructor who offers help to find the optimal exercise and conveys knowledge and confidence that the exercises performed are safe and effective. The characteristics of the instructor may play a role in influencing participants' attendance to exercise classes [42] and advisory support has previously been raised as important to strengthen participants' self-efficacy [43]. According to Bandura, the experience of mastery is the most important factor determining a person's self-efficacy, and in general people with high self-efficacy are more determined and persist longer than those with low self-efficacy [44]. In a particular activity confirmation may contribute to feelings of mastery or competence, which, according to the SDT, is important for successful behavior change [32, 33].

The participants in this study gave several examples of elements in the exercise they thought of as spirit lifters. These elements had the ability to provide energy and joy to the training and bring feelings of both vitality and relatedness. Outdoor activities were such spirit lifters. Recurrent preferences for outdoor exercises among Swedish seniors of all lifestyle categories have previously been reported [39, 45]. The importance of being outdoors and in contact with nature has been reported to enhance energy and induce greater vitality even when controlling for factors such as social interaction [46]. Maybe being outdoors could also contribute to important feelings of relatedness to the nature. Other spirit lifters mentioned by the participants were rhythm and music, exercise equipment, and humor. We believe all these elements may contribute to feelings of relatedness by creating a social context and building a bond between participants. However, feelings of relatedness were also seen in those who exercised alone. One example of this was how the use of walking poles created a sense of belongingness to a group of pole-walkers.

### 4.3. Methodological Considerations

A strength of this study was the design with multistage focus group discussions [14], which gave rich data through the process of meeting with community-dwelling participants on several occasions. The participants had the opportunity to reflect upon their exercise preferences, both during the focus group discussions and in between the sessions. It was sometimes difficult to know if the participants talked about physical activity in general, or about falls prevention in particular. However, all participants were aware that the study focused on exercise in the context of preventing falls and this was emphasized by the researchers during the discussions.

Another strength was the use of the PAAR methodology [17]. By encouraging informants to express themselves in a positive way, it has the potential to generate rich data about perceptions, feelings, experiences, motives, and attitudes. On the other hand, this appreciative approach may potentially result in an underestimation of negative thoughts and experiences, which might also be of importance. We believe that the appreciative approach of the PAAR methodology contributed to the friendly and positive atmosphere during the workshops. The attendance rate at all workshops was very high and the participants expressed that they liked coming to the meetings.

Yet another strength in this study was the sampling strategy, which gave variation in history of falls and experience of exercise in both men and women in each group. However, the transferability of the findings may be limited because most of the participants were retired white-collar workers, were in general well educated, and were mainly resource-rich with previous occupational experiences. Class differences in our study group were minor, which could have contributed to the similarity in exercise preferences. It should also be noted that participants in our study had enrolled in a fall preventive study with focus on exercising and thus shared a motivation for exercise. In this respect a similar interest may had a stronger influence than gender on the results.

The researchers involved were from different fields of expertise, physiotherapy, informatics, exercise physiology, and computing sciences, which enhanced both data collection and analysis [47]. The researchers brought experience from different methods and facilitating techniques that were applied during the workshops. The mix of methods helped the participants share their thoughts and contribute in various ways, which greatly enriched the data collection. Moreover, the author's diverse preunderstandings regarding falls prevention, balance and strength exercises, motivation for exercise, and gender research complemented each other and helped deepen the discussions within the author group, and thus the interpretation and analysis of the data.

The cultural context of the study could affect the generalizability of the results. As norms for how to be a man and a woman differ by age and cultural context, analysis of gender in older men and women needs to consider the wider context of norms for “being old” in the respective time and culture. In Sweden, older people are encouraged to stay active in order to avoid ill health. Investigations using accelerometers have shown that 49% between 65 and 75 years are physically active with a moderate or high intensity for at least 30 minutes during an average day, with very small gender differences [45]. The project was carried out in Umeå, Northern Sweden (latitude 63°N), where snow and icy roads are common in the winter period of November to April. Nature/forest is easily accessible, both in terms of walkability and of “legal rights” to walk everywhere/of public access. The results may thus not be generalizable to countries where the cultural norm of exercise, the gender norm, and attitudes to outdoor activities in old age differ.

## 5. Conclusions

Older people's preferences for exercise programmes are personal, and the individual differences are greater than the differences between men and women. Individually tailored exercise, in terms of mode, intensity, challenge, and social context, is important. In order to enhance uptake, adherence, and maintenance in fall prevention exercise professionals need to* see* the individual older person and apply autonomy-supportive approaches to encourage growth of intrinsic motivation. Besides individually tailored exercises such approaches should, according to older men and women, provide confirmational feelings, different spirit lifters to increase enjoyment, and personal tricks to maintain exercise routines. The study provides an awareness about women's and men's preferences for fall prevention exercises and this information could be used as guidance in designing individual tailored interventions that are inclusive, hence providing uptake and adherence for both sexes.

## Figures and Tables

**Table 1:** Characteristics of the participants.

**Code**	**Age**	**Focus group**	**Partner or living alone**	**White/Blue Collar**	**Falls last 12 months Yes/No**	**Physical activity level based on IPAQ**
**W1**	74	1	Partner	White	Yes	Moderate
**W2**	70	1	Partner	White	No	Moderate
**W3**	73	1	Partner	Blue	No	Moderate
**W4**	71	1	Partner	White	No	Moderate
**W5** ^**∗**^	76	1	Living alone	Blue	No	Moderate
**W6**	75	2	Living alone	White	No	Moderate
**W7**	80	2	Living alone	Blue	Yes	Low
**W8**	74	2	Partner	White	No	Moderate
**W9**	71	2	Partner	White	Yes	High
**W10**	70	2	Partner	White	No	Low
**M1**	72	1	Partner	White	Yes	Low
**M2**	80	1	Living alone	White	No	Moderate
**M3**	78	1	Partner	White	Yes	High
**M4**	76	1	Partner	White	No	Moderate
**M5**	70	2	Partner	White	No	Moderate
**M6**	79	2	Partner	White	No	Moderate
**M7**	75	2	Partner	White	No	Low
**M8**	79	2	Partner	White	Yes	Moderate

W: woman; M: man; IPAQ: International Physical Activity Questionnaire. White collar worker examples: office worker, police officer, nurse, and teacher; blue collar worker examples: taxi driver, shop keeper. The level of physical activity was self-reported as low, moderate, or high based on how much time had been spent being active the previous week, and how strenuous it had been. Examples of activities performed: cycling, swimming, water-aerobics, walks, table tennis, and weight lifting. ^*∗*^Dropped out after four focus group discussions.

## Data Availability

The interview data used to support the findings of this study are restricted by the Regional Ethical Review Board Umeå, Sweden, in order to protect patient privacy. Data are available from the corresponding author upon request.
